# Microbial diversity in individuals and their household contacts following typical antibiotic courses

**DOI:** 10.1186/s40168-016-0187-9

**Published:** 2016-07-30

**Authors:** Shira R. Abeles, Marcus B. Jones, Tasha M. Santiago-Rodriguez, Melissa Ly, Niels Klitgord, Shibu Yooseph, Karen E. Nelson, David T. Pride

**Affiliations:** 1 Department of Medicine, University of California, San Diego, 9500 Gilman Drive, MC 0612, La Jolla, CA 92093-0612 USA; 2 Human Longevity, Inc., San Diego, CA 92121 USA; 3 Department of Pathology, University of California, San Diego, 9500 Gilman Drive, MC 0612, La Jolla, CA 92093-0612 USA; 4 Genomic Medicine, J. Craig Venter Institute, La Jolla, CA 92037 USA

**Keywords:** Saliva, Gut, Skin, Microbiome, 16S rRNA, Antibiotic perturbations, Antibiotic courses, Antibiotics

## Abstract

**Background:**

Antibiotics are a mainstay of treatment for bacterial infections worldwide, yet the effects of typical antibiotic prescriptions on human indigenous microbiota have not been thoroughly evaluated. We examined the effects of the two most commonly prescribed antibiotics (amoxicillin and azithromycin) in the USA to discern whether short-term antibiotic courses may have prolonged effects on human microbiota.

**Results:**

We sampled the feces, saliva, and skin specimens from a cohort of unrelated, cohabitating individuals over 6 months. An individual in each household was given an antibiotic, and the other a placebo to discern antibiotic impacts on microbiota, as well as determine whether antibiotic use might reshape the microbiota of each household. We observed household-specific patterns of microbiota on each body surface, which persevered despite antibiotic perturbations. While the gut microbiota within an individual became more dissimilar over time, there was no evidence that the use of antibiotics accelerated this process when compared to household members. There was a significant change in microbiota diversity in the gut and mouth in response to antibiotics, but analogous patterns were not observed on the skin. Those who received 7 days of amoxicillin generally had greater reductions in diversity compared to those who received 3 days, in contrast to those who received azithromycin.

**Conclusions:**

As few as 3 days of treatment with the most commonly prescribed antibiotics can result in sustained reductions in microbiota diversity, which could have implications for the maintenance of human health and resilience to disease.

**Electronic supplementary material:**

The online version of this article (doi:10.1186/s40168-016-0187-9) contains supplementary material, which is available to authorized users.

## Background

The human body has many different surfaces and each is home to its own unique microbes [1], including cellular microbes (e.g., bacteria, archaea, and fungi) as well as large populations of viruses [2–6]. These microbes are collectively referred to as “the human microbiome,” and a litany of studies exist examining those microbes and their contributions to the maintenance of health and the development of disease [7–10]. Many microbiome studies focus on gut bacterial biota, which have been shown to be altered in conditions such as obesity [11–13], diabetes [14], and inflammatory bowel diseases [7, 15], and potentially may play roles in disorders of neurological development [16]. Host genetic factors, geography, and environmental variables (e.g., sex and hormonal fluctuations) have also been linked to microbes and their relative abundances in the human microbiome [17–22]. How human microbial communities respond to perturbations such as antibiotics, and whether such perturbations affect host susceptibility to disease, have become cornerstones of human microbiome research.

Microorganisms such as bacteria, fungi, viruses, and parasites cause many of the world’s diseases, yet only bacterial infections are usually susceptible to treatment with commonly prescribed antibiotics. The US Centers for Disease Control estimates that up to 50 % of prescribed antibiotics in the USA are unnecessary, and 30 % of those are prescribed for outpatients [23]. Their use has been associated with the emergence of antibiotic resistance, which resulted in an estimated 23,000 deaths in 2013. The Institute for Healthcare Informatics Review indicates that the two most overused antibiotics in the USA include azithromycin (56.3 million prescriptions annually in 2011) and amoxicillin (53.8 million prescriptions annually in 2011), compared to a population size of only 311.7 million in 2011. Despite the widespread use of azithromycin and amoxicillin, many studies characterizing the effects of antibiotics on the microbiome focus on other antibiotics such as clindamycin and ciprofloxacin [24, 25]. This focus in some cases is related to the commonly known effects of these drugs on human gut microbes, with ciprofloxacin having broad activity against *Proteobacteria* that inhabit the gut, and clindamycin known to alter gut microbiota in a manner that is closely associated with colonization and subsequent disease caused by *Clostridium difficile* [26, 27]. Very little is known about the effects of the azithromycin and amoxicillin (typically given in short 3- to 7-day courses) on the microbiota of human body surfaces, and whether their effects may be sustained over long time periods.

It has been established that cohabitating individuals share bacterial biota [28, 29]; however, in many of these studies, the cohabitating individuals had some genetic relationships. For example, monozygotic and dizygotic twins share a substantial proportion of their bacterial biota [21, 30], but do not necessarily need to cohabitate to have similarities in their microbiota. This phenomenon suggests that both proximity and host genetic factors contribute to the composition of the human microbiome. The sharing of microbiota between close contacts gains greater importance when considering that our microbiomes may carry antibiotic resistance [31, 32]. The potential for sharing antibiotic-resistant organisms in our microbiomes that may not be causing disease but could cause disease under certain circumstances is a growing public health concern. How or whether the sharing of our microbiota may be affected by the use of antibiotics has not previously been examined, as there may also be collateral effects of antibiotic use in an individual for their close contacts.

In this study, we recruited a cohort of 56 genetically unrelated individuals, with 48 of them living in pairs in 24 separate households. In each household, 1 individual took 3 to 7 days of an antibiotic and the other took 3 to 7 days of a placebo. Our goals were to (1) discern the effects of the 2 most commonly prescribed antibiotics on the microbiota of the skin, gut, and mouth, (2) characterize the degree of similarity in the microbiota of unrelated household contacts and decipher whether it is significantly affected by antibiotic use, (3) characterize the long-term effects of typical antibiotic prescriptions on microbiota diversity, and (4) discern whether there may be collateral effects to antibiotic use for the diversity of microbiota in household contacts.

## Results

### Study cohort

**Fig. 1:**
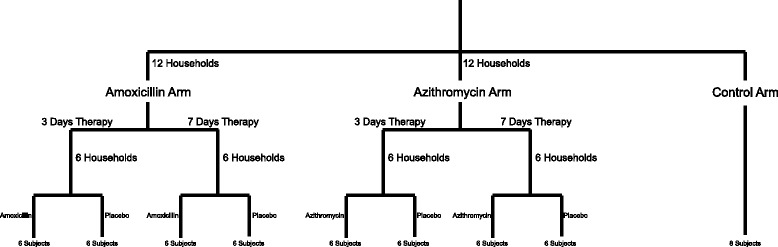
Flowchart of study design

We recruited and sampled the feces, saliva, and skin from a cohort of 56 subjects over a 6-month period from the University of California, San Diego, campus. Of those 56 individuals, there were 24 separate households consisting of 2 individuals and 8 separate controls not enrolled with a housemate (Fig. 1). In each household, 1 individual received treatment with an antibiotic (amoxicillin or azithromycin), and the other individual received treatment with a placebo (vitamin C). Twelve households received amoxicillin, with 6 households receiving 3 days and another 6 receiving 7 days of therapy. In these households, amoxicillin was given twice daily and the placebo also was given twice daily. Another 12 households received azithromycin, with 6 receiving 3 days and the other 6 receiving 7 days of therapy. The azithromycin was given once daily in these households, and the placebo was given once daily as well. The additional 8 subjects enrolled in the study were not enrolled with a housemate and did not receive antibiotic or placebo. Households were randomized into the separate arms of the study; however, the study subjects were not blinded because we had to account for existing drug allergies and medication interactions in our decisions to give them antibiotics. Study subjects were sampled on day 0 (day prior to antibiotics), day 3 (on the third day of antibiotics), day 7, week 8, and at 6 months.

There were no significant differences identified in the demographics of the subjects enrolled in the amoxicillin arm, azithromycin arm, or control arm of the study (Table 1). The mean age of subjects was 24 in the amoxicillin arm, 29 in the azithromycin arm, and 25 in the control arm. Approximately 50 % of the study participants were female, and none of the study subjects had taken systemic antibiotics for a year prior to the initiation of the study. The subjects lived together ranging from 1 to 54 months prior to the initiation of this study, and 58 % in the amoxicillin arm were romantic couples compared to 50 % in the azithromycin arm. The majority of study participants were Caucasian (54 %), with roughly equal numbers of Latino (25 %) and Asian subjects (21 %) (Additional file 1: Table S1). Five of the 24 households (2 in amoxicillin arm and 3 in azithromycin arm) were lost to follow-up after the 8-week time point and did not complete the 6-month time point.

**Table 1:** Demographics of study participants

Characteristics	Amoxicillin arm (*N* = 24)	Azithromycin arm (*N* = 24)	Control arm (*N* = 8)
Age (years)^¥^	24 ± 3.4	29 ± 13	25 ± 11
Female sex (%)	54	45	50
No. subjects with antibiotic consumption within last year	0	0	0
No. subjects with antibiotic consumption within last 3 years (%)	8 (33)	9 (37.5)	2 (25)
No. households who are couples (%)	7 (58)	6 (50)	NA
Approximate time living together prior to study (months)	16 (range 3 to 54)	16 (range 1 to 42)	NA

Plus-minus values indicate mean ± standard deviation

¥ No significant differences among groups by ANOVA

### Household-specific patterns in the mouth, gut, and skin

**Fig. 2:**
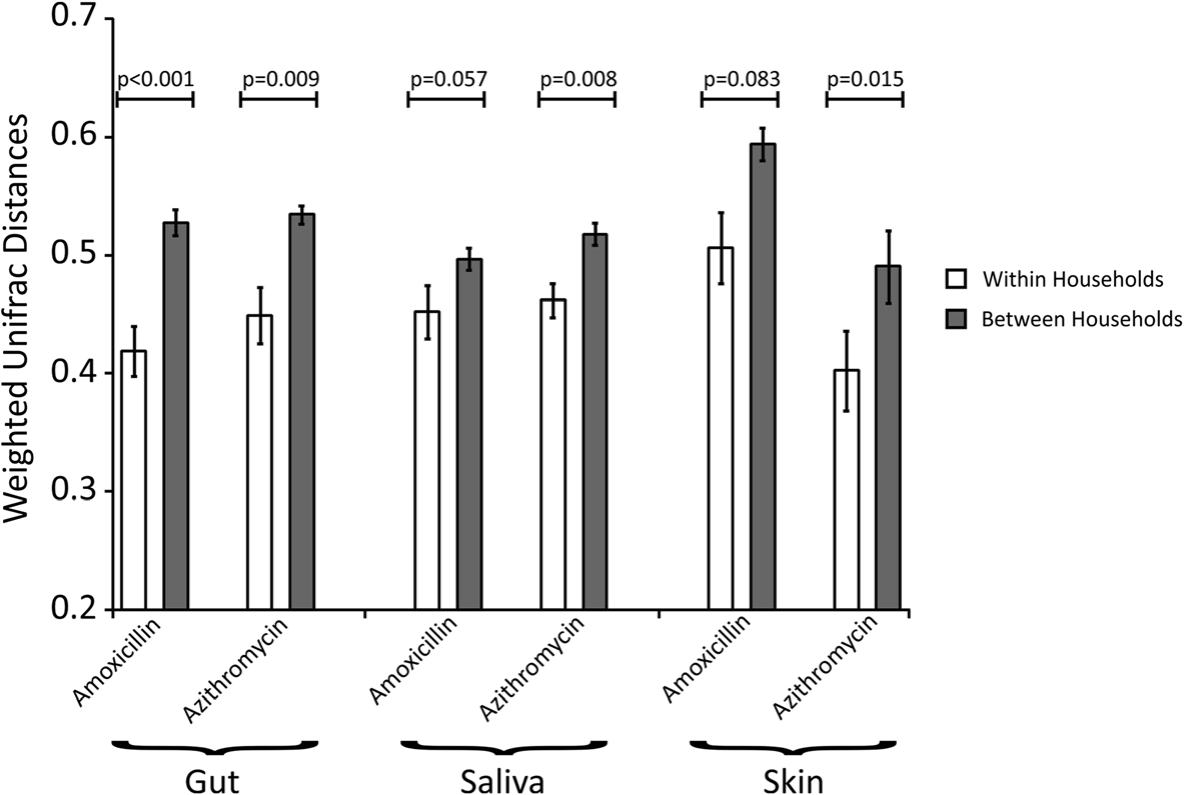
Bar graph representing mean weighted UniFrac distances (±standard error) within a household (*white bars*) and between different households (*gray bars*) based on the antibiotic received in each household. The *y*-axis represents mean weighted UniFrac distances, while the body site sampled and antibiotic received within each household is represented on the *x*-axis. *p* values were determined using the Mann Whitney *U* test

We sequenced the V1–V2 hypervariable segment of the 16S rRNA gene from the feces, saliva, and skin of all subjects at all time points in the study to characterize the bacterial biota present on each body surface with a total of 806 specimens (269 feces, 270 saliva, and 267 skin). Because previous studies that demonstrate household-specific patterns of bacterial biota are confounded by genetically related individuals living in those households [28, 29], we tested whether there were household-specific patterns in the bacterial biota among the genetically unrelated individuals in this cohort. By measuring weighted UniFrac distances [33] between household pairs longitudinally and comparing with individuals from separate households, we found smaller distances among the household pairs, which was statistically significant (*p* < 0.05) in the gut, saliva, and skin for all households (Additional file 2: Figure S1). When comparing household pairs with control subjects who were not enrolled with housemates, the distances were also significantly smaller in the gut and saliva, but not on the skin. The smaller distances indicate a higher degree of similarity among taxa and their relative abundances. The similarity observed in the bacterial biota was not significantly affected by the use of antibiotics, as the same patterns were observed in households that received azithromycin and those that received amoxicillin (Fig. 2). Additionally, the distances between subjects in each household was not significantly altered on any body surface over the course of the study (Additional file 2: Figure S2), suggesting that the use of antibiotics did not substantially change the collective microbiota of the household.

We measured weighted UniFrac distances among the households based on whether the individuals in the households were romantic couples or roommates to discern whether personal relationships significantly shape the shared microbiota within the household. We found that in all households, there was a statistically significant pattern of shared microbiota (*p* < 0.05 in each case; data not shown); however, there was no significant relationship identified in the feces, saliva, or skin based on whether the individuals were couples or roommates. There was a trend toward more shared taxa in couples, but the trend was not statistically significant on any body surface (Additional file 2: Figure S3 and S4).

### Effects of time on microbiota compositions

**Fig. 3:**
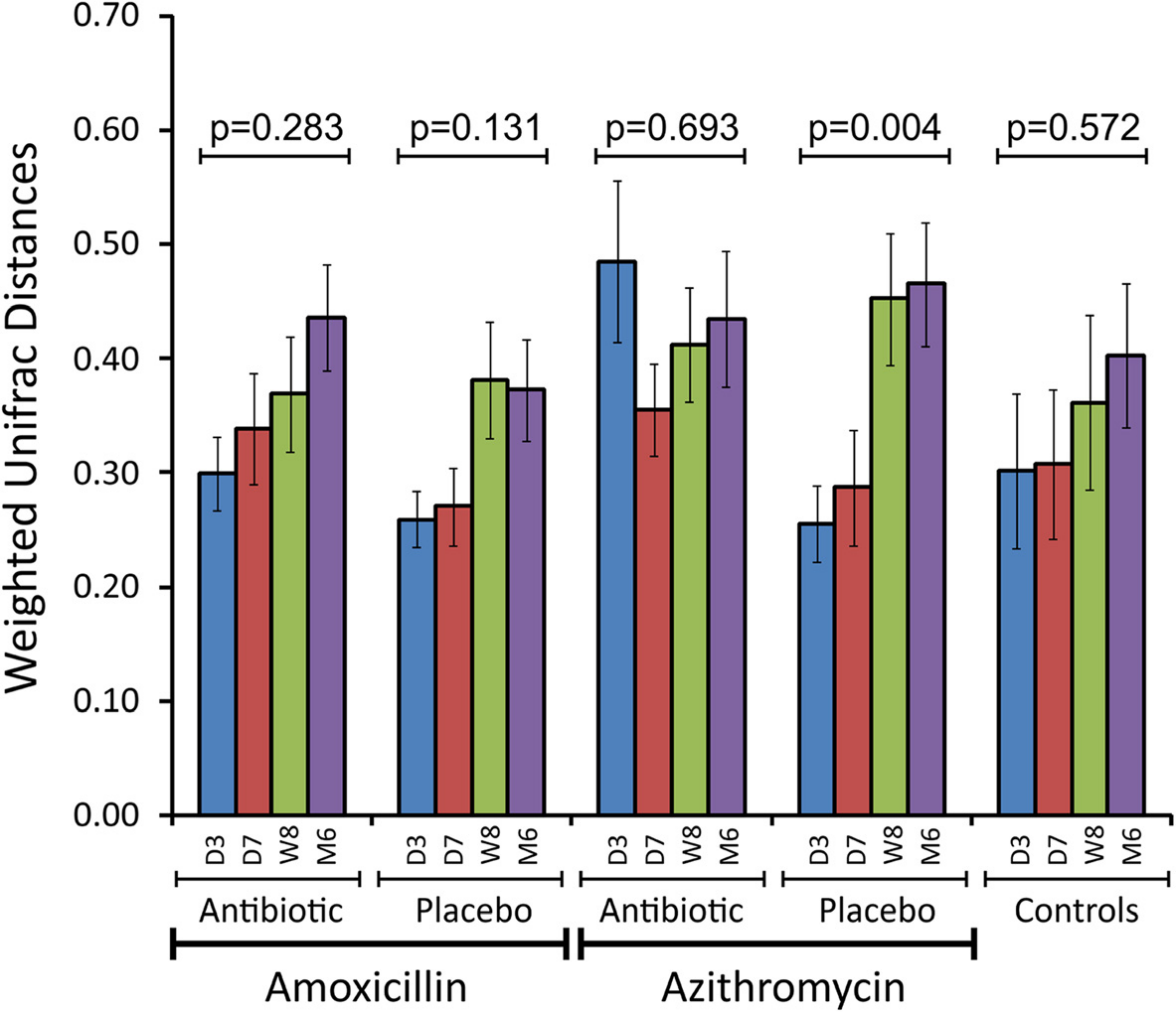
Bar graph (±standard error) representing the mean weighted UniFrac distances from day 0 in the feces of all subjects over time. The *y*-axis represents mean weighted UniFrac distances, and the *x*-axis represents the different subject groups over time based on the therapy they received. *D3* represents day 3, *D7* represents day 7, *W8* represents week 8, and *M6* represents month 6. *p* values were determined using the Kruskal Wallis test

We evaluated whether microbiota compositions within subjects were becoming more dissimilar over time, and whether the use of antibiotics may accelerate this process. We measured weighted UniFrac distances between day 0 (day prior to antibiotics or placebo) and each subsequent time point to identify whether the degree of dissimilarity increased over time. In the gut, the microbiota of subjects taking amoxicillin and azithromycin generally grew more dissimilar over each time point measured (Fig. 3). While these data were not statistically significant, a clear trend could be observed for most time points. For those subjects taking placebo, the exact same trend was identified. This trend was not a direct result of the placebo or antibiotic therapy, as those control subjects who received no treatment also demonstrated the same trend. The magnitude of the dissimilarity among the different antibiotic, placebo, and control groups did not differ significantly. These data suggest that while the gut microbiota grow more dissimilar within a subject over time, the use of short courses of common antibiotics does not significantly accelerate the process. No similar trends were identified in the mouth (Additional file 2: Figure S5) or on the skin (Additional file 2: Figure S6), suggesting that the gut is unique among these body surfaces in the evolution of its microbial communities.

### Taxonomic responses to antibiotics

**Fig. 4:**
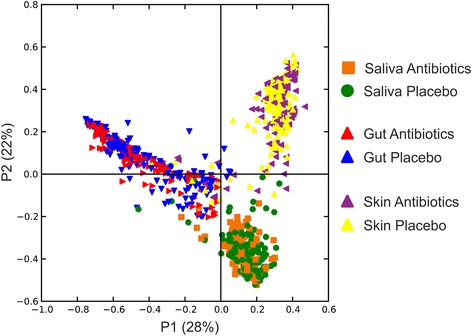
Principal coordinates analysis of beta diversity present in all subjects, time points, and sample types based on whether they received antibiotics (amoxicillin or azithromycin) or placebo

We examined the taxonomic compositions of all subjects across the different body surfaces over time to decipher whether there were differences attributable to the antibiotics. We first measured beta diversity within and between all subjects and visualized the output using principal coordinates analysis (Fig. 4). Most of the samples reflected the body site from which they were derived and could not be distinguished based on whether or not the subject received an antibiotic or placebo.

**Fig. 5:**
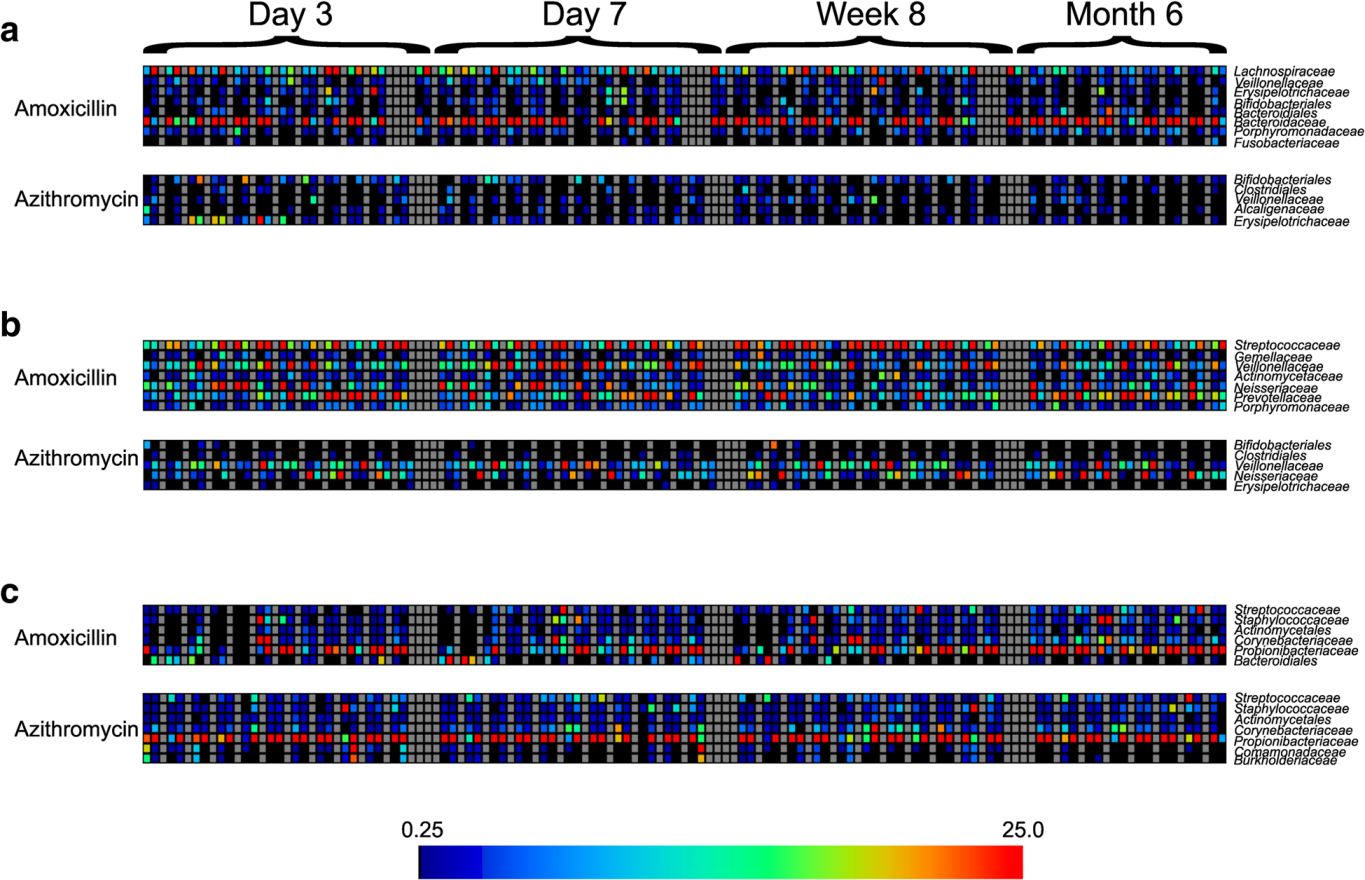
Heatmaps representing the relative abundances of taxa in individuals taking antibiotics that were significantly different when compared to their housemates receiving placebo. Each individual taking an antibiotic is shown next to their housemate taking a placebo. Each household consisting of two subjects is separated by *gray vertical boxes*. **a** Feces, **b** saliva, and **c** skin. The family or order for each OTU shown on the heatmaps is shown to the *right* of each heatmap, and the antibiotic received is shown to the *left* of each heatmap. The index color scale is shown below

In the gut, the most abundant taxa identified belonged to families *Bacteroidaceae*, *Lachnospiraceae*, and *Ruminococcaceae*. In both the azithromycin and amoxicillin arms of the study, the abundance of *Lachnospiraceae* was significantly diminished after antibiotic therapy and remained diminished at 6 months (Additional file 2: Figure S7). We compared the taxa from each individual taking antibiotics with their housemate taking a placebo to decipher whether there were taxa in each household whose relative abundance was altered as a response to the antibiotics. *Lachnospiraceae* were significantly diminished (*p* ≤ 0.01) in response to amoxicillin on days 3 and 7 in most households, but their relative abundances increased significantly in comparison to their housemates by week 8 (Fig. 5). *Veillonellaceae*, *Bacteroidales*, and *Porphyromonadaceae* (anaerobic bacteria) were significantly decreased in response to amoxicillin, while *Fusobacteriaceae* (also anaerobes) were increased. *Bifidobacteriales* and *Erysipelotrichaceae* were initially decreased, and subsequently increased in comparison to their housemates taking placebo. In response to azithromycin therapy, *Erysipelotrichaceae*, *Veillonellaceae*, and *Clostriales* were significantly diminished, while *Alcaligenaceae* were increased compared to their housemates.

In the saliva, the most abundant taxa identified were *Prevotellaceae*, *Streptococcaceae*, *Veillonellaceae*, and *Neisseriaceae* (Additional file 2: Figure S7). In response to amoxicillin, *Veillonellaceae*, *Actinomycetaceae*, *Neisseriaceae*, *Prevotellaceae*, and *Porphyromonadaceae* were all significantly increased in comparison to their housemates, while *Streptococcaceae* and *Gemellaceae* were diminished (Fig. 5). In response to azithromycin, *Bifidobacteriales* and *Veillonellaceae* were increased, while *Clostridiales*, *Neisseriaceae*, and *Erysipelotrichaceae* were diminished in comparison to their housemates.

On the skin, the most abundant taxa identified were *Corynebacteriaceae*, *Propionibacteriaceae*, *Staphylococcaceae*, and *Streptococcaceae* (Additional file 2: Figure S7). In response to both amoxicillin and azithromycin, the relative abundances of *Streptococcaceae*, *Staphylococcaceae*, *Actinomycetales*, *Corynebacteriaceae*, and *Propionibacteriaceae* were all altered in comparison to their housemates (Fig. 5).

### Reduction in microbial diversity

**Fig. 6:**
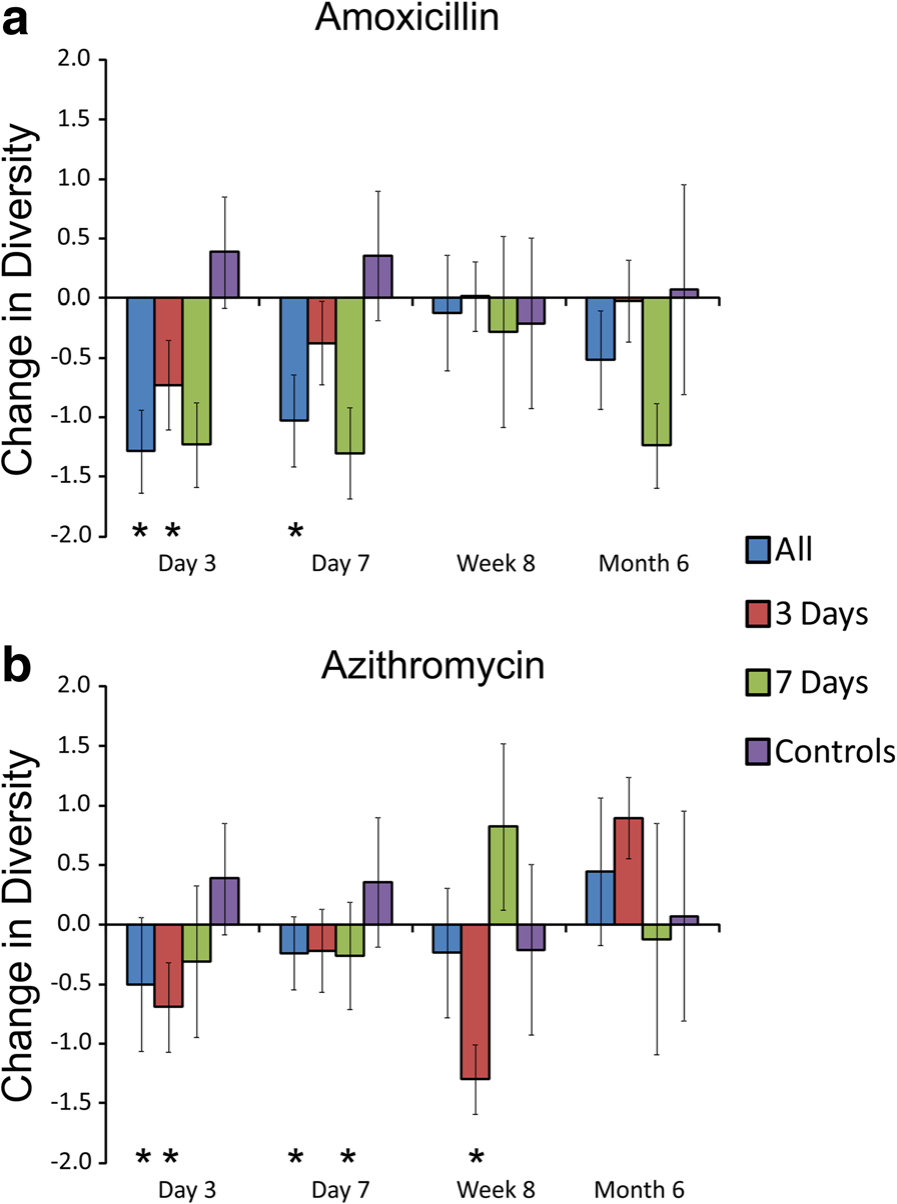
Bar graphs (±standard error) representing the normalized difference in Shannon diversity in the gut between individuals taking antibiotics and their housemates taking placebo at each time point studied. **a** Households that took amoxicillin and placebo and **b** households that took azithromycin and placebo. All households collectively are represented by the *blue bars*, households that took 3 days of an antibiotic are represented by *red bars*, households that took 7 days of an antibiotic are represented by *green bars*, and control subjects who were not enrolled with housemates and are represented by *purple bars*. The *x*-axis represents the time point and the *y*-axis represents the change in normalized change in Shannon diversity since the prior time point. Negative results indicate lower diversity in the subjects taking antibiotics compared to their housemates taking placebo, and positive results indicate greater diversity in the housemates taking placebo compared to the individuals taking antibiotics. For the control subjects, the *bars* represent the mean change in diversity among all control subjects. *p* values were determined using the Mann Whitney *U* test, and *p values ≤0.05

**Fig. 7:**
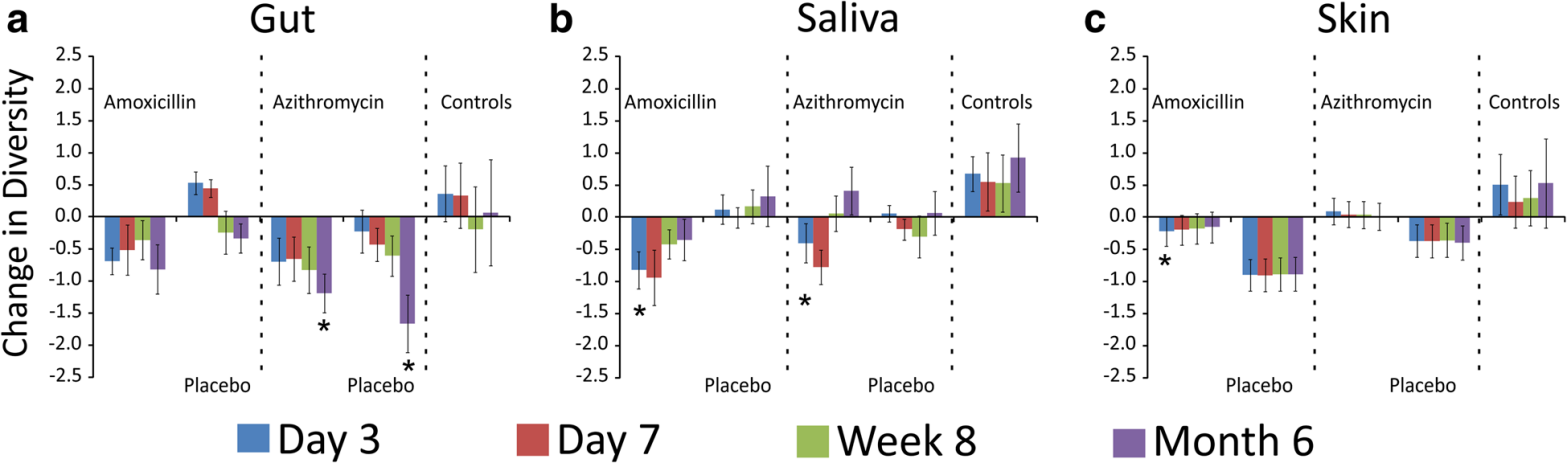
Bar graphs (±standard error) representing the change in Shannon diversity from day 0 across time in each subject group by body site tested. **a** Feces, **b** saliva, and **c** skin. Groups that received antibiotics, placebo, or no therapy (controls) are labeled across the *x*-axis, and the *y*-axis represents the change in Shannon diversity. *p values <0.05 using the Mann Whitney *U* test comparing subject groups at specified time points with the controls

We characterized the diversity of the microbial communities on each body surface in response to antibiotics to decipher whether diversity was substantially impacted longitudinally by the use of typical antibiotic courses. We first examined changes in microbiota diversity in each subject compared to their housemate taking a placebo. In the gut, we found that there was a significant reduction in diversity compared to their housemates for subjects taking amoxicillin (Fig. 6a). While there was a trend toward a sustained reduction in microbiota diversity compared to their housemates at 8 weeks and 6 months, the data were not statistically significant. Reductions in diversity were observed in subjects taking only 3 days of amoxicillin, but they were generally less than those observed in subjects who took 7 days of amoxicillin. Similar results were identified when comparing subjects who took azithromycin with their housemates. At 3 and 7 days, significant reductions in gut microbial diversity was observed, but this reduction was not sustained throughout the study (Fig. 6b). There were no significant differences observed between subjects who took 3 or 7 days of azithromycin. We also analyzed reductions in gut diversity in subjects who took amoxicillin and azithromycin and compared to control subjects rather than their housemates (Fig. 7a). We found that there was a substantial reduction in microbiota diversity in subjects who took either amoxicillin or azithromycin and that those reductions were sustained throughout the 6-month study. Interestingly, we also identified reductions in the diversity of their housemates, which was not observed in control subjects. When examining changes in diversity time point by time point, the majority of the diversity reductions occurred within the first 3 days of antibiotic therapy (Additional file 2: Figure S8).

**Fig. 8:**
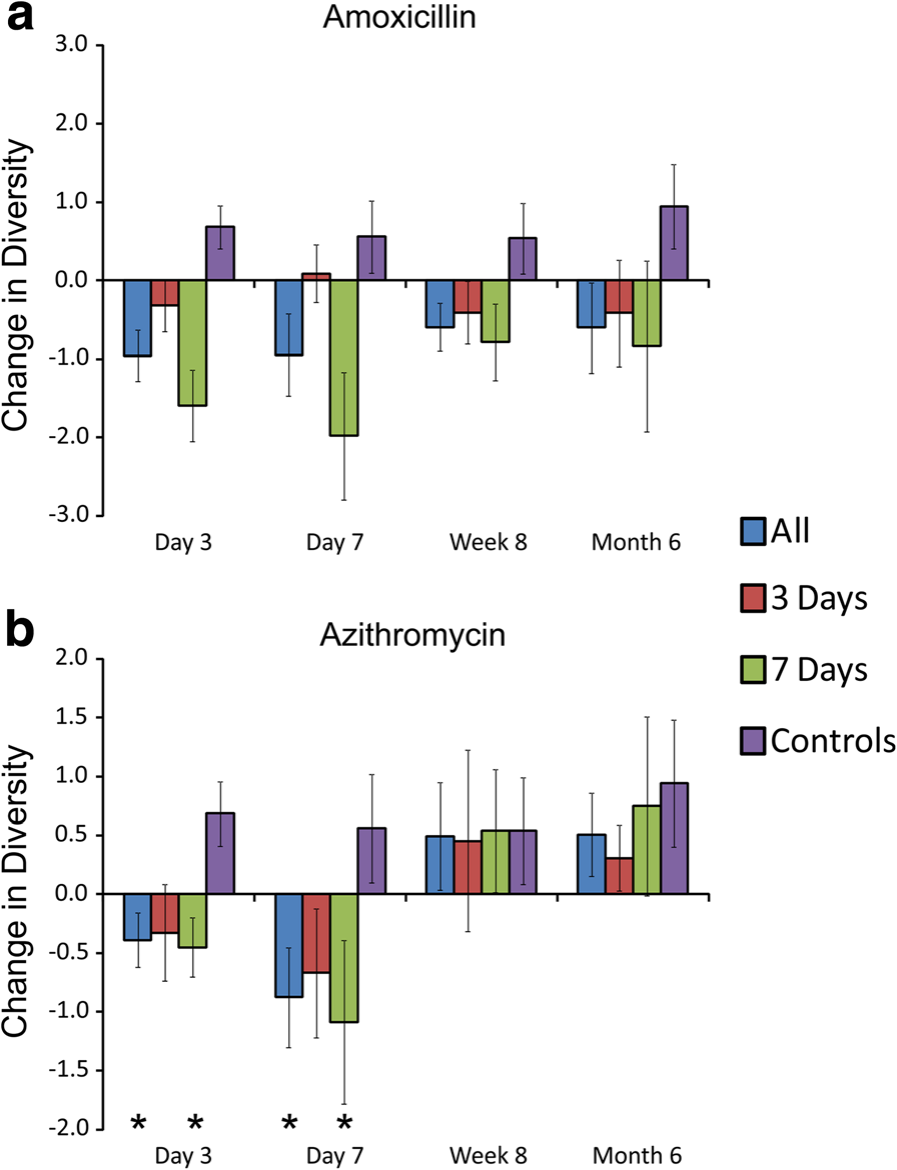
Bar graphs (±standard error) representing the normalized difference in Shannon diversity in the saliva between individuals taking antibiotics and their housemates taking placebo at each time point studied. **a** Households that took amoxicillin and placebo and **b** households that took azithromycin and placebo. All households collectively are represented by the *blue bars*, households that took 3 days of an antibiotic are represented by *red bars*, households that took 7 days of an antibiotic are represented by *green bars*, and control subjects who were not enrolled with housemates and are represented by *purple bars*. The *x*-axis represents the time point and the *y*-axis represents the change in normalized change in Shannon diversity since the prior time point. Negative results indicate lower diversity in the subjects taking antibiotics compared to their housemates taking placebo, and positive results indicate greater diversity in the housemates taking placebo compared to the individuals taking antibiotics. For the control subjects, the *bars* represent the mean change in diversity among all control subjects. *p* values were determined using the Mann Whitney *U* test, and *p values ≤0.05

We also compared changes in oral microbiota diversity between the subjects within a household. We identified reductions in microbiota diversity in subjects taking amoxicillin compared to their housemates, but most reductions were not statistically significant (Fig. 8a). As was found in the gut, there were generally greater reductions in microbiota diversity in response to 7 days of amoxicillin than were observed after just 3 days. In response to azithromycin, we found significant reductions in microbiota diversity after both 3 and 7 days of therapy, but those reductions were not sustained by week 8 (Fig. 8b). We also examined reductions in microbiota diversity independent of their housemates, and found that there were sustained reductions in microbiota diversity in response to amoxicillin over the entire 6 months of the study, but that reductions in diversity in response to azithromycin were not sustained (Fig. 7b). Unlike the reductions in microbiota diversity in the guts of housemates who took a placebo, there were no significant diversity reductions in the oral microbiota of housemates. The majority of the diversity reductions that took place in response to antibiotics occurred within the first 3–7 days of therapy (Additional file 2: Figure S8).

When compared to housemates, subjects who received amoxicillin saw a reduction in their cutaneous microbiota diversity after 3 days, but not in response to azithromycin (Additional file 2: Figure S9). Independent of their housemates, this reduction in microbiota diversity in response to amoxicillin was sustained over the 6 months of the study (Fig. 7c). We also observed a reduction in diversity of the subjects who received the placebo, which was greater than that observed in response to antibiotics. The reduction in microbiota diversity observed in response to amoxicillin occurred within the first 3 days of therapy (Additional file 2: Figure S8).

## Discussion

We sought to elucidate the effects of the most commonly prescribed antibiotics in the USA on the microbiota of human body surfaces. Because antibiotics often are absorbed across the GI tract and distributed to the tissues via the bloodstream, we expected that each would affect the microflora of each body surface tested. Both amoxicillin and azithromycin often are prescribed unnecessarily, and their effects on the microbiota of each body surface may have implications for resilience to pathogen colonization and susceptibility to disease. We found that each antibiotic had significant impacts upon the microbiota of the mouth and gut that were apparent after only 3 days of therapy (Additional file 2: Figure S8). Other studies have demonstrated that lesser prescribed antibiotics such as ciprofloxacin, clindamycin, and minocycline also can have profound and lasting effects on the microbiota of the gut [24, 25].

Bacterial biota can be shared between individuals in a household, and those household-specific patterns observed on body surfaces also extend to household surfaces such as floors and doorknobs [29]. We did not recruit any subjects with known genetic relationships in an attempt to avoid confounders that may result in similar microbiota between individuals. On all body surfaces studied, we could identify household-specific patterns using weighted UniFrac distances, which take into account both the presence of taxa and their relative abundances. When this same analysis was performed using unweighted distances that do not account for relative abundances, the data were no longer significant (data not shown), which suggests that relative abundances of taxa on various body surfaces are influenced by the household environment. Unfortunately, the individuals in this study had been cohabitating for various time periods prior to the initiation of this study, so the rapidity with which these household-specific patterns developed could not be assessed. We also identified a trend of greater similarity among the microbiota of romantic couples compared to roommates, similar to that which has previously been described in the oral cavities of couples [34]. While we did not record information on physical contact between housemates, we believe the greater similarity in microbiota among romantic couples reflected greater physical contact. Despite the substantial conservation of the gut microbiota within subjects and households over time, genetic distances increased over time within each subject [35], indicating that there was some turnover of gut taxa and/or sustained alterations in their relative abundances over time. Interestingly, this trend did not appear to be accelerated by the use of antibiotics (Fig. 3), which suggests that this trend would have taken place even without the interventions that took place in this study.

By studying the two most commonly prescribed antibiotics in the USA, we wanted to identify whether there were sustained effects of common antibiotic treatments on the human microbiome. Our use of multiple controls allowed for us to discern whether microbiota diversity was significantly impacted compared to close contacts and to strangers. Because the external controls were not enrolled in the study with housemates and did not receive either antibiotic or placebo, we believe that they provided the most reliable reference group with regard to microbiota variation over time. We found separate trends in our study depending on which controls we used, which highlights that there may be collateral effects to antibiotic use for our close contacts. For example, there were significant diversity reductions in gut microbiota in response to amoxicillin and azithromycin compared to close contacts, but those reductions were not sustained after 8 weeks (Fig. 6). This trend largely reflects the concomitant reduction in gut microbiota diversity that is observed in the housemates of those taking antibiotics that was not identified in the other control subjects (Fig. 7). We originally hypothesized that we would observe an increase in gut microbiota diversity due the sharing of microbiota with housemates and did not predict that we would observe diversity reductions in the housemates. These data suggest that the influence of close contact on an individual’s microbiome may extend beyond simply sharing microbes and may indicate that there is a balance in microbe sharing that contributes to diversity.

We observed different responses of host microbiota based on both the antibiotic used and the length of therapy. While these antibiotics are known to have different spectrums of activity against microbes, they also differ significantly in their half-life, which might result in different effects on our microbiota. Amoxicillin has a half-life of 1 h, while azithromycin has a half-life of 68 h. Thus, we might expect the effects of shorter courses of azithromycin to have similar effects as longer courses because the drug will remain in the host for an extended period regardless of whether the subject receives a 3- or a 7-day course. This is what we observed in both the feces and saliva of subjects taking azithromycin (Figs. 6b and 8b ). We observed the opposite trend in subjects taking amoxicillin. Because the drug is eliminated from subjects much more rapidly, we believe that we observed more profound effects on diversity in those taking a 7-day course than in those taking a 3-day course (Figs. 6a and 8a).

Prior to this study, we believed that the use of these common antibiotics would accelerate microbiota changes significantly over 6 months. We found that the subjects who took antibiotics were no less similar to their original state than were subjects who took placebo or no therapy at all (Fig. 3 and Additional file 2: Figure S5 and S6). We did, however, identify changes in microbiota diversity that did not recover to baseline regardless of the antibiotic taken (Fig. 7). We did not follow the subjects beyond 6 months, so we cannot rule out the possibility that the differences we observed in diversity after just 3 to 7 days of antibiotics could render these subjects more susceptible to pathogen colonization.

Similar to a prior study, there was a near complete recovery of microbial diversity in the saliva [25] regardless of the antibiotic that was used (Fig. 7b). In that study, minocycline, amoxicillin, clindamycin, and ciprofloxacin were used and each showed a similar trend. We utilized a placebo in this study to simulate the model of a clinical trial and initially intended for the study to be blinded. We unblinded the study because many of the study participants were taking oral contraceptives, which would interact with azithromycin. The placebo (vitamin C) could have affected the microbiota of the skin, as the subjects who received vitamin C had substantial diversity decreases in their skin microbiota (Fig. 7c). Because vitamin C is rapidly excreted when high plasma levels are reached, we did not expect to observe significant microbiota changes after only 3 to 7 days of placebo.

## Conclusions

Our data help to illustrate important concepts regarding the responses of our microbiota to common antibiotic perturbations. The shared microbiota in the gut, mouth, and on the skin of close contacts who were not known to be genetically related bolsters the concept that genetic relationships are not required to share our microbiota. We believe that the close contact and shared environmental reservoirs resulted in the sharing of microbiota within the households; however, there are other confounders which could also affect the relative proportions of shared microbiota within a household. For example, 14 of the 24 households studied had pets including dogs, cats, fish, and hamsters (Additional file 1: Table S1), which may also have served as vehicles for the sharing of microbiota. We recorded specific details regarding diet preferences for each subject studied, but did not note any differences between the subject groups that might account for the microbiota differences observed (data not shown). Only two of the subjects studied were vegetarians (Additional file 1: Table S1), but their microbiota was more similar to their housemates than to each other. The impact of short typical antibiotic courses persisted for at least 6 months in the gut, a factor that should be taken into account whenever antibiotics are prescribed. While our cohort was not followed nearly long enough to ascertain whether there may be risks to the sustained diversity reductions, the concomitant reductions in diversity observed in housemates suggests that antibiotic prescriptions may pose collateral risks to our close contacts.

## Methods

### Cohort design

Forty-eight subjects were enrolled in the study in pairs, with 2 individual living in each household. An additional 8 individuals were enrolled without a housemate and received no therapy over the course of the 6-month study. Households were randomized into either the amoxicillin or azithromycin arms of the study. Those subjects also were randomized to receive either antibiotic or placebo; however, because of the large numbers of penicillin allergies reported (Additional file 1: Table S1) and subjects using oral contraceptives (interact with azithromycin), some subjects who were randomized to receive antibiotics were given the placebo, while their housemate received the antibiotic instead. Of the household pairs, 6 pairs were placed into the 3-day amoxicillin arm, 6 pairs were placed into the 7-day amoxicillin arm, 6 pairs were placed into the 3-day azithromycin arm, and 6 pairs were placed into the 7-day azithromycin arm (Fig. 1). In each household, 1 subject received either 3 or 7 days of an antibiotic and the other subject received either 3 or 7 days of the placebo (vitamin C). The dose of amoxicillin was 500 mg twice daily, and the dose of vitamin C was 500 mg twice daily. The dose of azithromycin was 500 mg on the first day and 250 mg daily thereafter (this dosing was used to be consistent with the commonly prescribed Z-Pak). In the azithromycin arm, the placebo was given at 500 mg once daily. Each subject enrolled donated saliva, feces, and a skin swab on day 0 (day prior to antibiotics), day 3 (3 days after initiation of antibiotics), day 7, week 8, and month 6. Of the 24 households enrolled, 5 of those households (Additional file 1: Table S1) were lost to follow-up and did not provide specimens at the month 6 time point. Each subject provided at least 3 ml of unstimulated saliva and a skin swab from the volar surface of the forearm (the same forearm surface was used for each sampling throughout the study) [36–38]. Skin swabs were immediately placed into a solution of 0.15 M NaCl and 0.1 % Tween 20 [37]. All subjects were encouraged to provide specimens in the AM prior to breakfast and freeze them at −20 °C prior to transporting on ice to the study site, where they were frozen at −80 °C until use in this study. Exclusion criteria included prior antibiotic use for 1 year prior to the initiation of the study and preexisting medical conditions such as diabetes, inflammatory bowel disease, and organ transplantation that might result in significant immunosuppression. All subjects self-reported their health status and were genetically unrelated.

### Analysis of 16S rRNA

Genomic DNA was prepared from the saliva and skin swabs of each subject and time point using the QIAGEN QIAamp DNA MINI kit (QIAGEN). Each sample was subjected to a bead beating step prior to nucleic acid extraction using Lysing Matrix-B (MP Bio). Genomic DNA was prepared from fecal samples using the QIAGEN QIAamp DNA Stool Mini Kit (QIAGEN). We amplified the bacterial 16S rRNA gene V1–V2 hypervariable region using the forward primer 8F (AGAGTTTGATCCTGGCTCAG) fused with the Ion Torrent Adaptor A sequence and 1 of 70 unique 10 base pair barcodes and reverse primer 357R (CTGCTGCCTYCCGTA) fused with the Ion Torrent Adaptor P1 from each donor and sample type [39]. PCR reactions were performed using Platinum High Fidelity PCR SuperMix (Invitrogen) with the following cycling parameters: 94 °C for 10 min, followed by 30 cycles of 94 °C for 30 s, 53 °C for 30 s, 72 °C for 30 s, and a final elongation step of 72 °C for 10 min. Resulting amplicons were purified on a 2 % agarose gel stained with SYBR Safe (Invitrogen) using the MinElute PCR Purification Kit (QIAGEN). Amplicons were further purified with Ampure XP beads (Beckman-Coulter), and molar equivalents were determined for each sample by quantifying the amplicons using PicoGreen (Invitrogen) using a plate reader. Samples were pooled into equal molar proportions and sequenced on 316 chips using an Ion Torrent PGM according to manufacturer’s instructions (Life Technologies) [40]. Resulting sequence reads were removed from the analysis if they were <180 nucleotides or >500 nucleotides, had any barcode or primer errors, contained any ambiguous characters, or contained any stretch of >8 consecutive homopolymers. Sequences then were trimmed according to any site that had a Phred score of less than 10 [41]. Sequences then were assigned to their respective samples based on a 10-nucleotide barcode sequence and were further processed to remove reads that were greater than 3 standard deviations from the mean read lengths in any specimen. Skin reads were further processed to remove any reads matching >97 % to a read from an *Alphaproteobacteria* identified from the uninoculated control solution using Ion Assist (www.thepridelaboratory.org).

We sequenced a minimum of 10,000 reads from each sample and analyzed the sequence data using Quantitative Insights Into Microbial Ecology (QIIME 1.5) [42]. We randomly sampled 8000 reads from each sample to create subsamples that were used in each analysis using Ion Assist (www.thepridelaboratory.org). Representative OTUs from each set were chosen at a minimum sequence identity of 97 % using the QIIME script pick_otus_through_otu_table, which uses the Greengenes database [43]. PCOA was performed based on beta diversity using weighted UniFrac distances [33] using the QIIME script beta_diversity_through_plots. The results of the beta diversity distance matrices were used to determine the weighted UniFrac distances between different samples and sample groups. For each subject group, we determined weighted UniFrac distances between each subject pair within a household at each time point studied and compared those distances with between all pairs of subjects who did not reside in the same household at the same time points. Statistical significance was determined by the Mann Whitney *U* test using MaxStat Pro (www.maxstat.de). This technique was utilized for determining the distances between household pairs who were couples or roommates, household pairs who received amoxicillin and placebo, and household pairs who received azithromycin and placebo. We also calculated the distances between all household pairs receiving a specific therapy at each time point studied. Statistical significance was performed by comparing the mean distances over time among household pairs using the Kruskal Wallis test using MaxStat Pro. We utilized this technique to determine distances over time among households who received amoxicillin and azithromycin or that contained couples or roommates.

Alpha diversity using the Shannon diversity index [44] was determined using QIIME. The results were normalized by body site to allow for direct comparisons of changes in diversity between all the study subjects. We used several different techniques for comparing diversity between the study subjects. First, we calculated the change in diversity for all subjects by comparing each time point with their Shannon Index values on day 0. For each household, we next compared the change in diversity between the subjects by subtracting the change in diversity of the subject taking the placebo from the change in diversity observed in the subject taking antibiotics. Negative values indicated that there was a reduction in diversity in the subjects taking antibiotics compared to their housemate, and positive values indicated that there was greater diversity in subjects taking antibiotics compared to their housemate. The change in diversity values were calculated for all household pairs in different subject groups, and the means and standard errors utilized to decipher trends. Statistical differences in the means were determined using the Mann Whitney *U* test. We utilized this technique to decipher whether subject groups who received amoxicillin and azithromycin had significant differences in diversity between the housemates. The next technique that we utilized was to determine whether there were diversity reductions present independent of housemates. We first normalized the Shannon index values by body site and calculated the change in diversity using day 0 as the index value. We calculated this change in diversity among all subjects who received amoxicillin, azithromycin, placebo, or no therapy and utilized the means and standard errors to help decipher trends in the data. Statistical significance was determined by comparing each time point between groups who received antibiotics with those who received no therapy by the Mann Whitney *U* test. The last technique utilized did not use day 0 as an index time point and instead compared the change in diversity across each time point studied. Statistical differences in alpha diversity were determined using the Mann Whitney *U* test for comparisons between each group taking antibiotics and those of the controls.

We identified OTUs with significant changes in their relative abundances by examining the OTU tables produced using QIIME. These OTU tables were normalized and visually represented as heatmaps utilizing MEV 4.9 [45]. Significant differences (*p* < 0.01) in individual taxa were determined by direct comparisons of each subject taking antibiotics with their housemate. These comparisons were performed using the Rank Products algorithm [46] typically used to identify differential expression in response to perturbations. We reported only those taxa that were significantly different between household members and represented 1 % or greater of the taxa present in the majority of individuals studied.

## Additional files

Additional file 1: Table S1. Study subjects. (PDF 275 kb) Additional file 2: This file contains Figures S1–S9. (PDF 1247 kb)
