# Supplementary material for: Microbial diversity in individuals and their household contacts following typical antibiotic courses
Source: Microbiome. 2016 Jul 30;4:39. doi: 10.1186/s40168-016-0187-9 (PMC4967329; doi:10.1186/s40168-016-0187-9)
Supplement: Additional file 1: Table S1. — Study subjects. (PDF 275 kb) [file 40168_2016_187_MOESM1_ESM.pdf]

Table S1: Study Subjects

Subjects	Household	Antibiotic	Roommate or Couple	Days of therapy	Ethnicity	Age	Sex	Dropped out at 6 months	Side Effects or Complications	Household Pet	Vegetarian	Medication Allergies
CA05	a	Amoxicillin	Couple	7	Caucasian	23	F	No	None	None	No	None
CA06	a	Placebo	Couple	7	Caucasian	24	M	No	None		No	Penicillin
CA07	b	Amoxicillin	Couple	7	Asian	23	M	Yes	None	None	No	None
CA08	b	Placebo	Couple	7	Asian	22	F	Yes	None		No	Penicillin
CA09	c	Amoxicillin	Roommate	3	Caucasian	26	M	No	None	4 dogs	No	None
CA10	c	Placebo	Roommate	3	Caucasian	22	M	No	None		Yes	None
CA11	d	Azithromycin	Couple	7	Asian	19	F	Yes	None	None	No	None
CA12	d	Placebo	Couple	7	Latino	20	M	Yes	None		No	None
CA13	e	Amoxicillin	Couple	3	Caucasian	31	F	No	None	1 dog	No	None
CA14	e	Placebo	Couple	3	Caucasian	27	F	No	None		No	None
CA15	f	Azithromycin	Roommate	7	Asian	24	M	Yes	None	None	No	None
CA16	f	Placebo	Roommate	7	Asian	21	M	Yes	None		No	None
CA17	g	Amoxicillin	Couple	3	Asian	19	M	No	None	None	No	None
CA18	g	Placebo	Couple	3	Asian	19	F	No	None		Yes	None
CA19	h	Amoxicillin	Roommate	3	Caucasian	24	M	No	None	1 cat	No	Sulfa
CA20	h	Placebo	Roommate	3	Caucasian	26	M	No	None		No	Penicillin
CA21	i	Amoxicillin	Roommate	7	Latino	22	F	Yes	None	1 cat	No	None
CA22	i	Placebo	Roommate	7	Asian	21	F	Yes	None		No	None
CA23	j	Amoxicillin	Couple	7	Caucasian	34	M	No	None	1 dog	No	None
CA24	j	Placebo	Couple	7	Caucasian	21	M	No	None		No	Penicillin
CA25	k	Amoxicillin	Roommate	3	Latino	22	F	No	None	1 hamster	No	None
CA26	k	Placebo	Roommate	3	Latino	23	F	No	None		No	None
CA27	l	Amoxicillin	Couple	7	Caucasian	24	M	No	None	None	No	None
CA28	l	Placebo	Couple	7	Caucasian	24	F	No	None		No	Penicillin
CA29	m	Amoxicillin	Roommate	7	Caucasian	23	F	No	None	None	No	None
CA30	m	Placebo	Roommate	7	Caucasian	23	F	No	None		No	None
CA31	n	Amoxicillin	Couple	3	Caucasian	27	M	No	None	None	No	None
CA32	n	Placebo	Couple	3	Caucasian	26	F	No	None		No	None
CA33	o	Azithromycin	Roommate	3	Latino	26	F	No	None	None	No	None
CA34	o	Placebo	Roommate	3	Caucasian	45	F	No	None		No	Penicillin
CA35	p	Azithromycin	Couple	3	Latino	22	F	No	None	2 dogs	No	None

CA36	p	Placebo	Couple	3	Latino	24	M	No	None		No	None
CA37	q	Azithromycin	Roommate	7	Caucasian	21	M	No	Mild diarrhea	1 cat	No	None
CA38	q	Placebo	Roommate	7	Caucasian	22	M	No	None		No	None
CA39	r	Azithromycin	Roommate	7	Asian	63	M	No	None	None	No	None
CA40	r	Placebo	Roommate	7	Latino	60	M	No	None		No	None
CA43	t	Azithromycin	Couple	3	Caucasian	21	F	No	None	2 dogs	No	None
CA44	t	Placebo	Couple	3	Caucasian	21	M	No	None		No	None
CA47	v	Azithromycin	Couple	7	Caucasian	24	M	No	None	1 dog	No	None
CA48	v	Placebo	Couple	7	Caucasian	25	F	No	None		No	None
CA49	w	Azithromycin	Couple	3	Caucasian	26	M	Yes	None	1 dog	No	None
CA50	w	Placebo	Couple	3	Caucasian	26	F	Yes	None		No	None
CA53	y	Azithromycin	Couple	7	Caucasian	20	F	No	None	2 cats	No	None
CA54	y	Placebo	Couple	7	Caucasian	21	M	No	None		No	None
CA55	z	Azithromycin	Roommate	3	Latino	28	F	No	None	1 dog	No	None
CA56	z	Placebo	Roommate	3	Latino	25	F	No	None		No	None
CA57	aa	Azithromycin	Roommate	3	Latino	30	F	No	None	2 fish	No	None
CA58	aa	Placebo	Roommate	3	Latino	57	M	No	None		No	None
CA02	None	None	Neither	0	Latino	20	F	No	None	1 dog	No	None
CA81	None	None	Neither	0	Caucasian	19	F	No	None	None	No	None
CA83	None	None	Neither	0	Asian	20	M	No	None	1 dog	No	None
CA84	None	None	Neither	0	Caucasian	24	M	No	None	None	No	None
CA85	None	None	Neither	0	Asian	21	M	No	None	1 dog	No	None
CA86	None	None	Neither	0	Caucasian	51	M	No	None	None	No	None
CA87	None	None	Neither	0	Latino	28	F	No	None	None	No	None
CA89	None	None	Neither	0	Asian	19	F	No	None	None	No	None
