# Supplementary material for: Microbial diversity in individuals and their household contacts following typical antibiotic courses
Source: Microbiome. 2016 Jul 30;4:39. doi: 10.1186/s40168-016-0187-9 (PMC4967329; doi:10.1186/s40168-016-0187-9)
Supplement: Additional file 2: — This file contains Figures S1–S9. (PDF 1247 kb) [file 40168_2016_187_MOESM2_ESM.pdf]

Figure S1

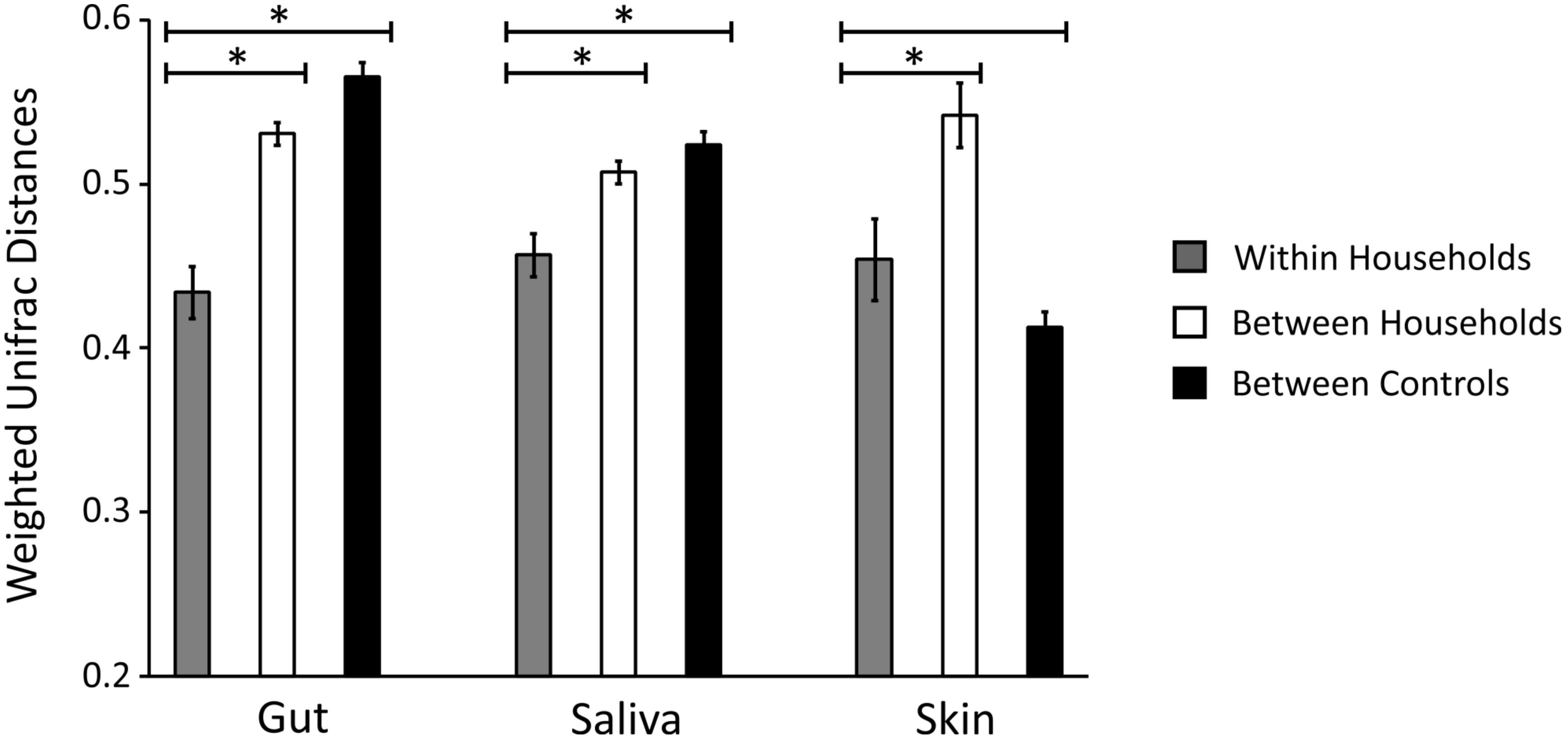

Figure S1: Bar graph representing mean weighted UniFrac distances (± standard error) within a household (white bars), between different households (gray bars), and amongst the control subjects (black bars) who were not enrolled with household members. The y-axis represents mean weighted UniFrac distances, and the body site sampled is represented on the x-axis. p-values were determined using the Mann Whitney U test, and the '*' represents p-values ≤0.05.

Figure S2

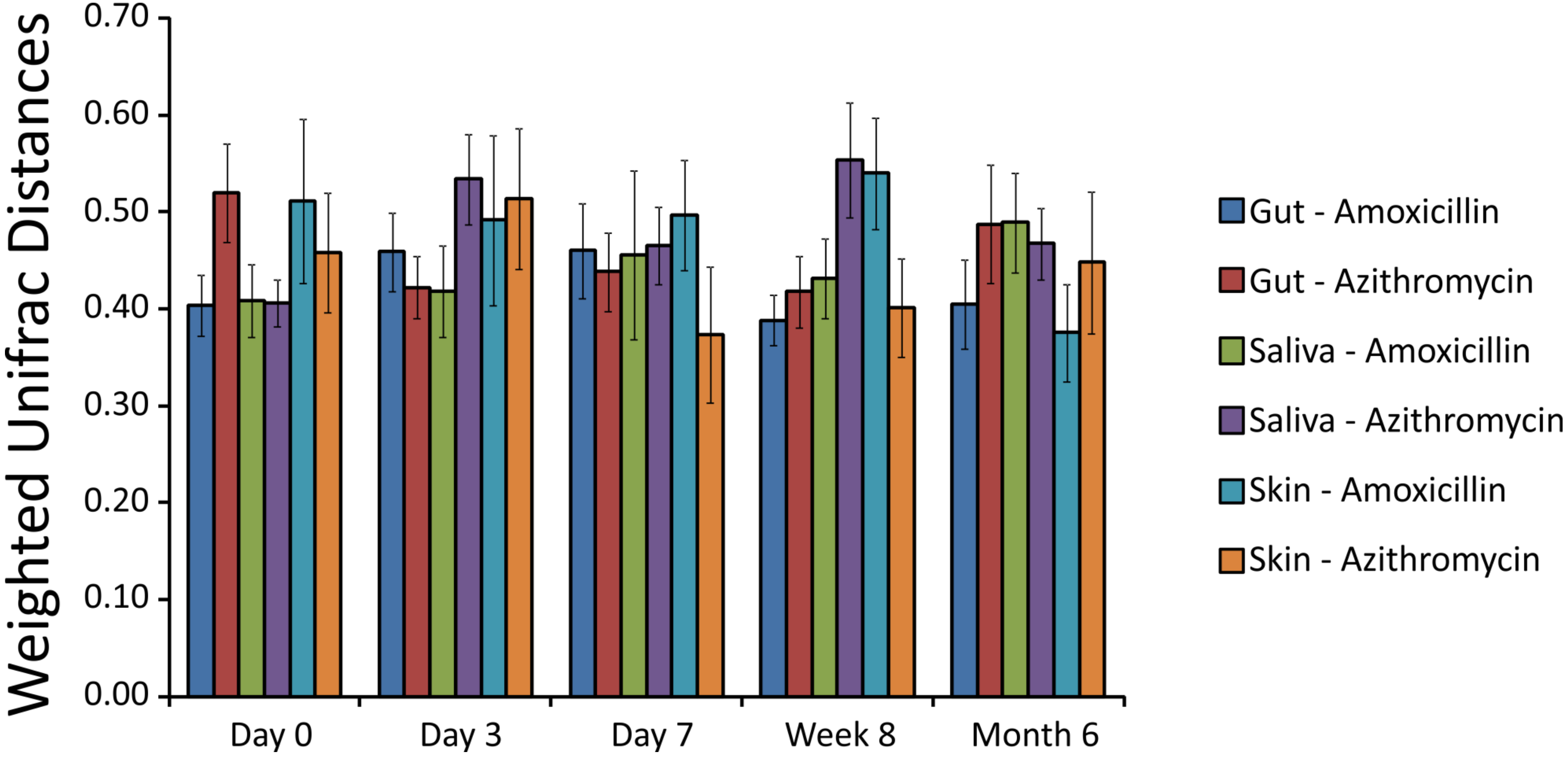

Figure S2: Bar graphs representing mean weighted UniFrac distances (± standard error) between housemates who received antibiotics and placebo over time. The y-axis represents mean weighted UniFrac distances, and the time point being compared is represented on the x-axis.

Figure S3

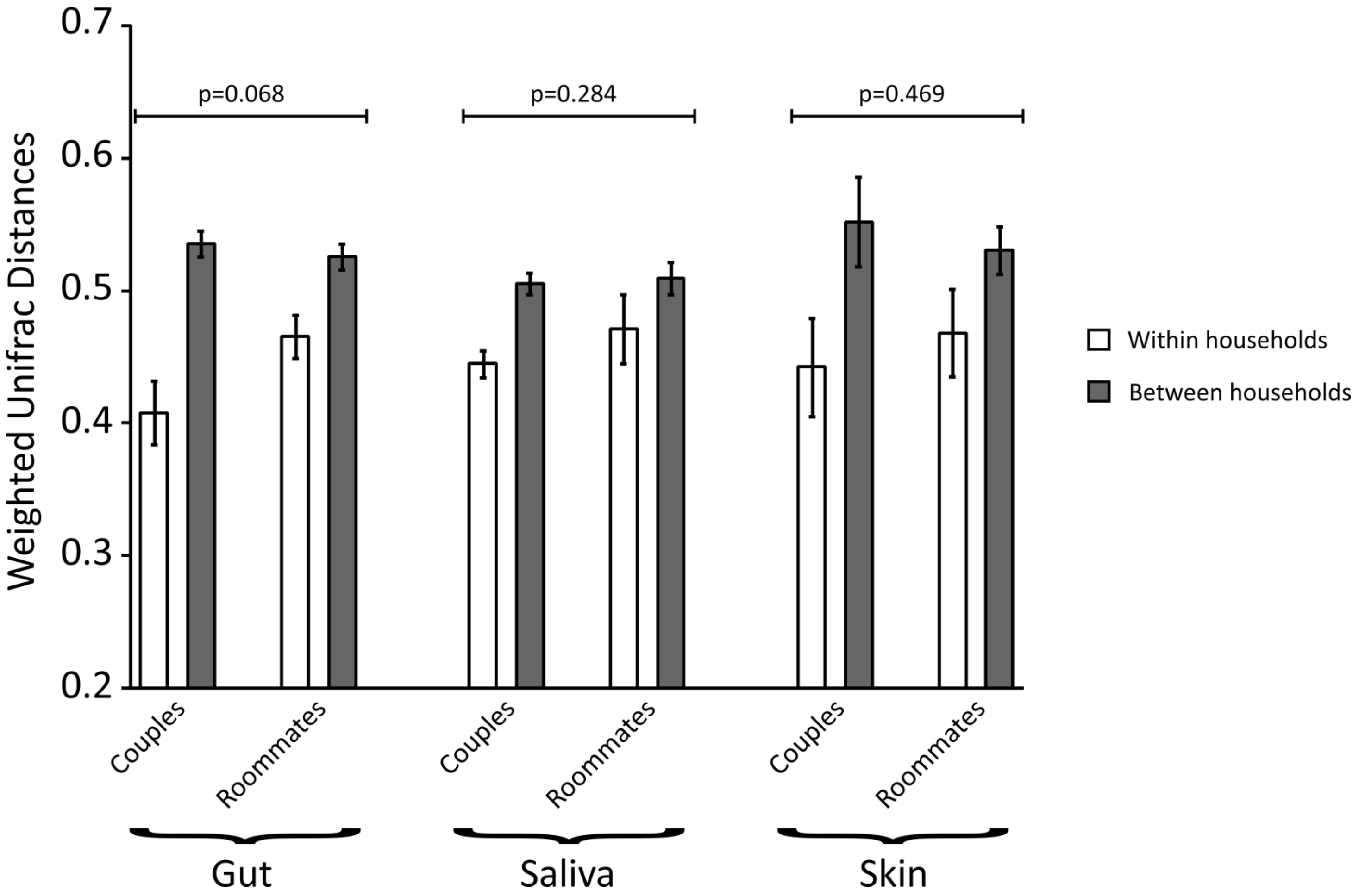

Figure S3: Bar graph representing mean weighted UniFrac distances (± standard error) within a household (white bars) and between different households (gray bars) based on whether the housemates were couples or roommates. The y-axis represents mean weighted UniFrac distances, and the body site sampled is represented on the x-axis. p-values represent comparisons within households of couples vs roommates, and were determined using the Mann Whitney U test.

Figure S4

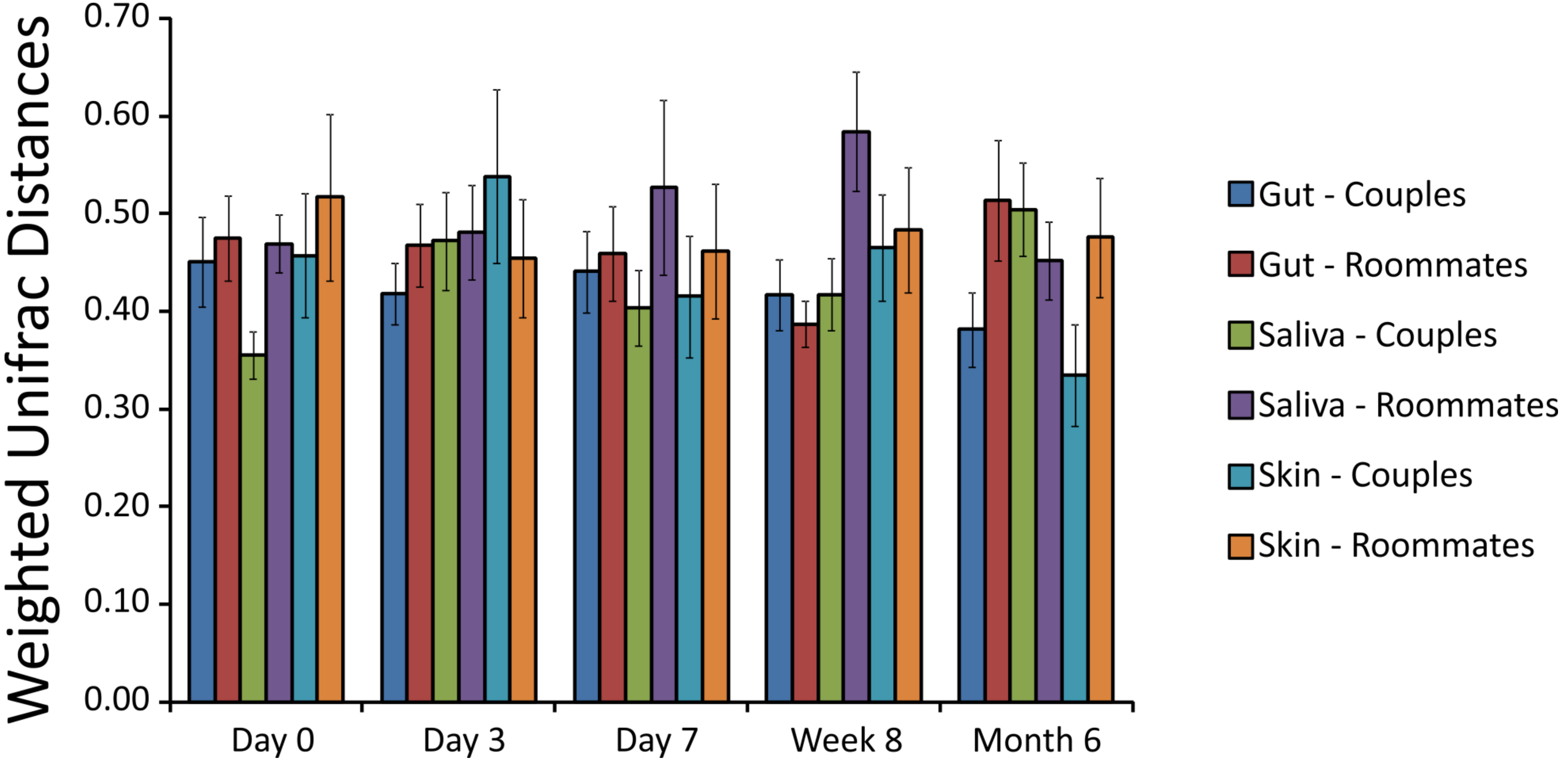

Figure S4: Bar graphs representing mean weighted UniFrac distances (± standard error) between housemates who were couples or roommates over time. The y-axis represents mean weighted UniFrac distances, and the time point being compared is represented on the x-axis.

Figure S5

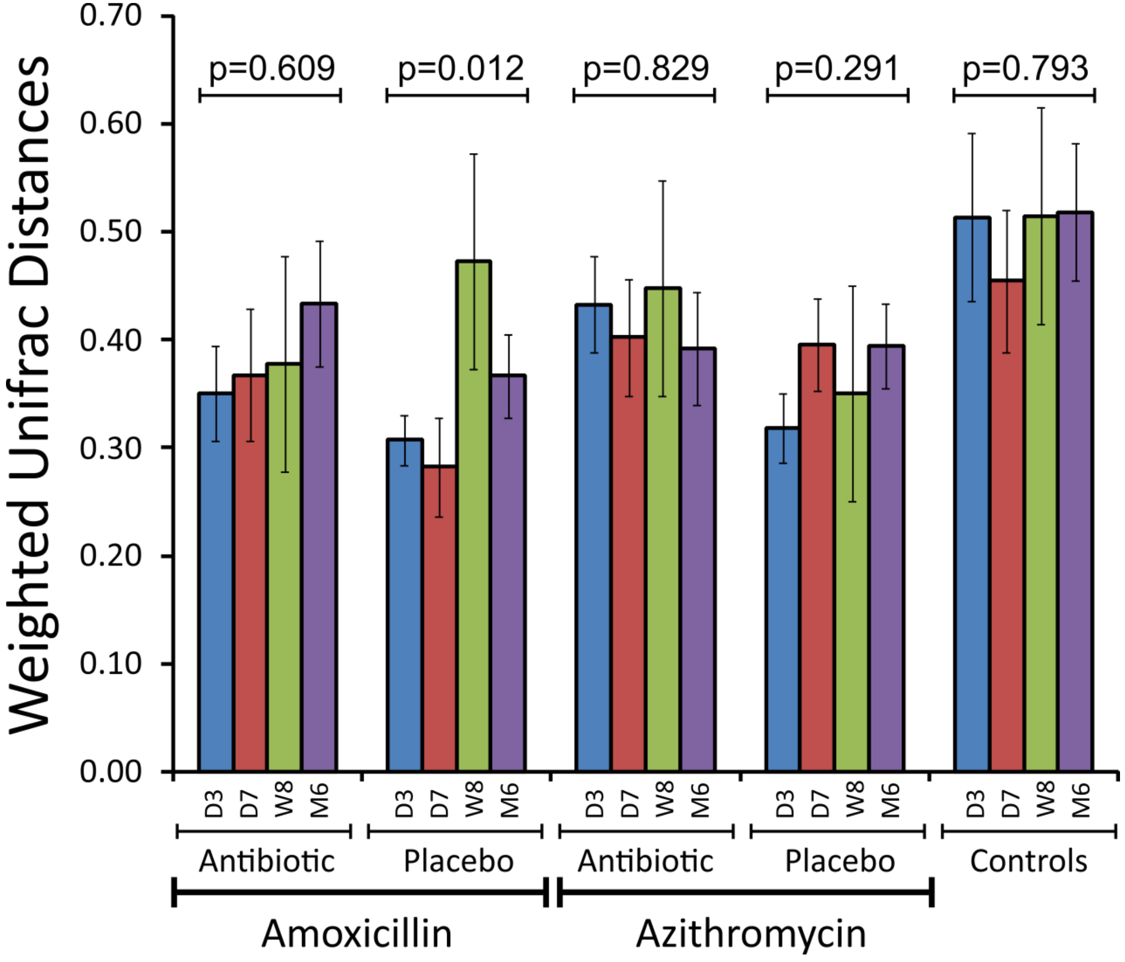

Figure S5: Bar graph (± standard error) representing the mean weighted UniFrac distances from day 0 in the saliva of all subjects over time. The y-axis represents mean weighted UniFrac distances, and the x-axis represents the different subject groups over time based on the therapy they received. D0 represents day 0, D3 represents day 3, D7 represents day 7, W8 represents week 8, and M6 represents month 6. p-values were determined using the Kruskal Wallis test.

Figure S6

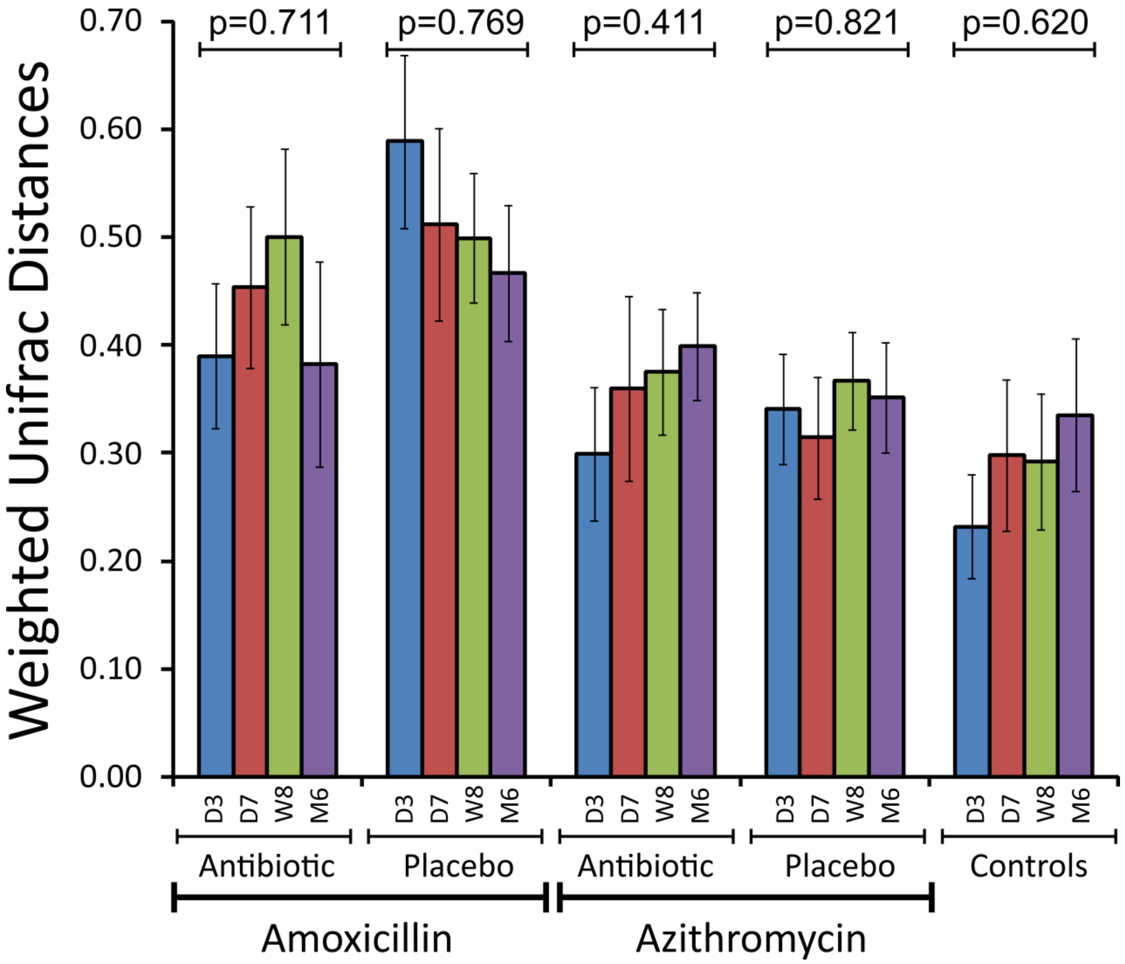

Figure S6: Bar graph (± standard error) representing the mean weighted UniFrac distances from day 0 on the skin of all subjects over time. The y-axis represents mean weighted UniFrac distances, and the x-axis represents the different subject groups over time based on the therapy they received. D0 represents day 0, D3 represents day 3, D7 represents day 7, W8 represents week 8, and M6 represents month 6. p-values were determined using the Kruskal Wallis test.

Figure S7

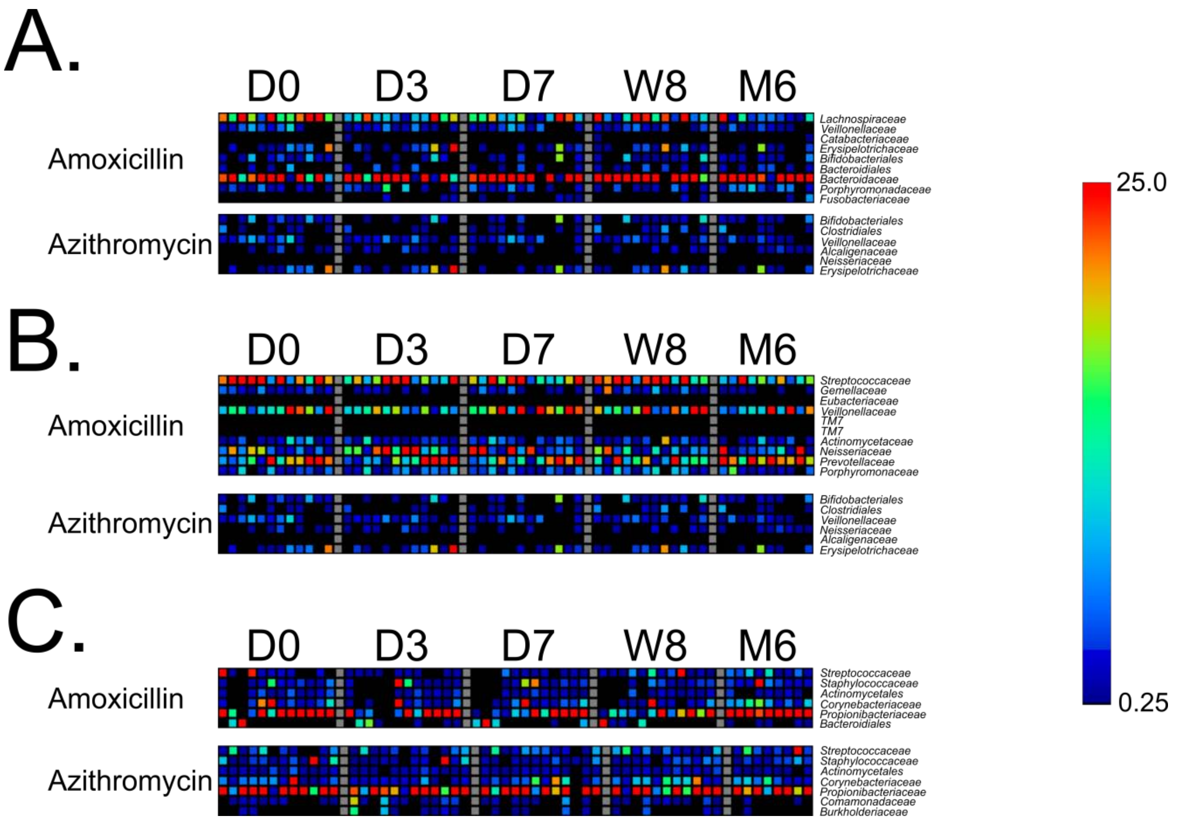

Figure S7: Heatmaps representing the relative abundances of taxa in individuals taking antibiotics that were significantly different when compared to their housemates receiving placebo. Panel A represents feces, Panel B represents saliva, and Panel C represents skin. D0 represents day 0, D3 represents day 3, D7 represents day 7, W8 represents week 8, and M6 represents month 6. Each subject is represented from left to right across the columns on the heatmap where the first column for D0 represents the relative abundances of taxa in a subject on day 0 and the first column for D3 represents the relative abundances at day 3 for that same subject. The family or order for each OTU shown on the heatmaps is shown to the right of each heatmap, and the antibiotic received is shown to the left of each heatmap. The index color scale is shown on the right.

Figure S8

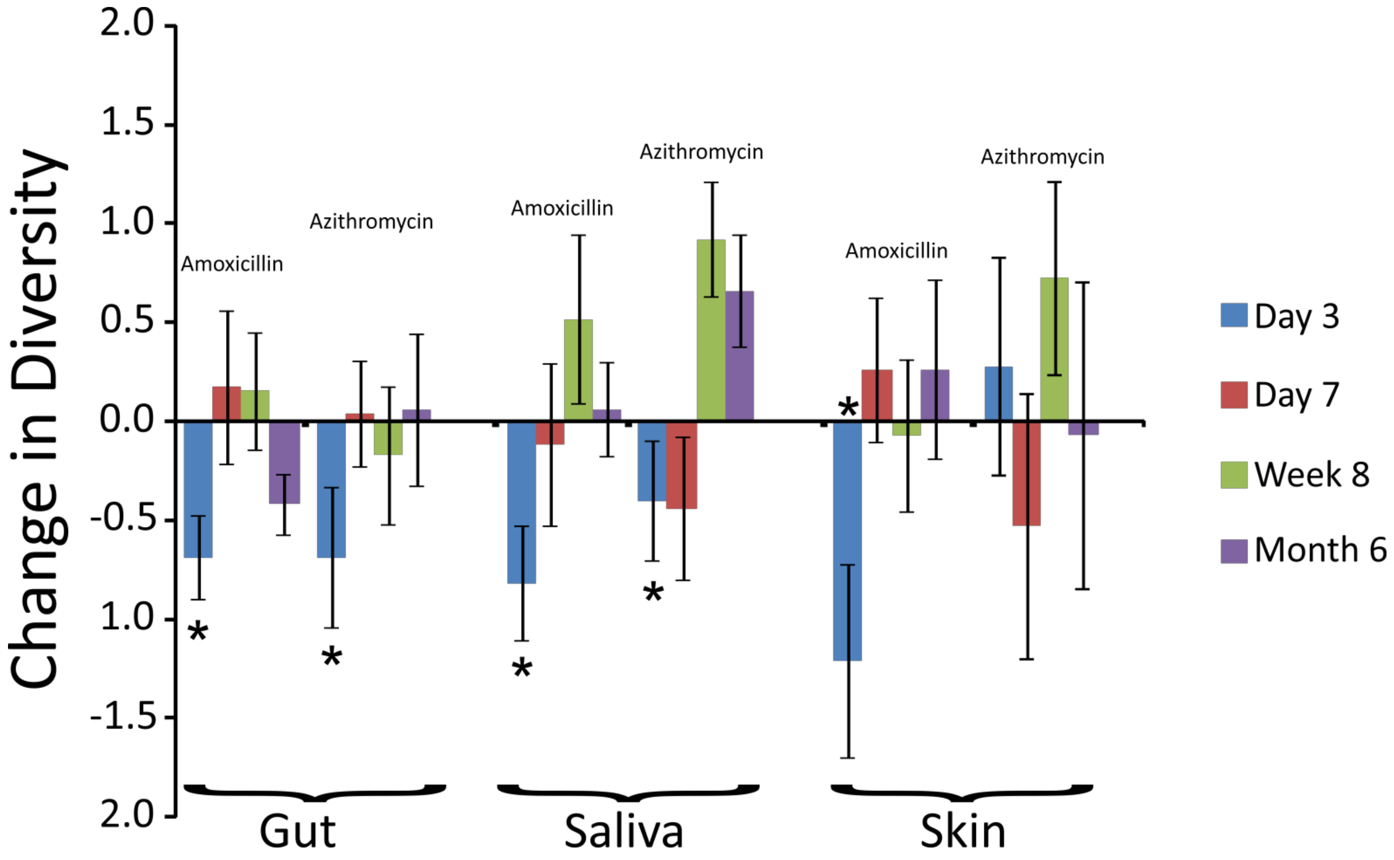

Figure S8: Bar graphs (± standard error) representing the change in Shannon diversity across time in each subject group by body site tested. Change in diversity is measured as differences in diversity between each individual time point. The y-axis represents the change in Shannon diversity between time points, and the x-axis represents the sample type and the antibiotic received. The '*' indicates p-values <0.05 determined using the Mann Whitney U test comparing subject groups with controls at each time point.

Figure S9

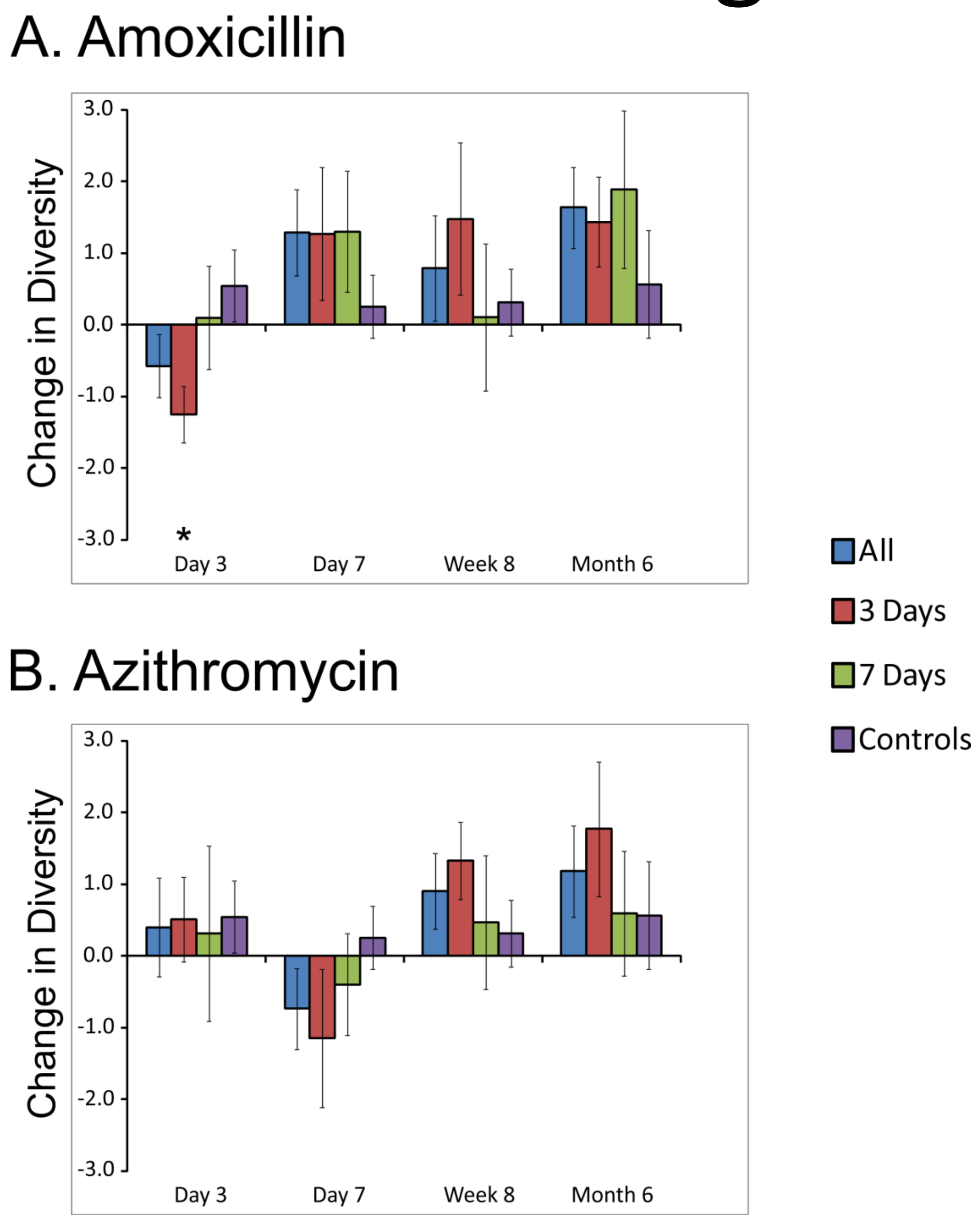

Figure S9: Bar graphs (± standard error) representing the normalized difference in Shannon diversity on the skin between individuals taking antibiotics and their housemates taking placebo at each time point studied. Panel A represents households that took amoxicillin and placebo and Panel B represents households that took azithromycin and placebo. All households collectively are represented by the blue bars, households that took 3 days of an antibiotic are represented by red bars, households that took 7 days of an antibiotic are represented by green bars, and control subjects who were not enrolled with housemates and are represented by purple bars. The x-axis represents the time point and the y-axis represents the change in normalized change in Shannon diversity since the prior time point. Negative results indicate lower diversity in the subjects taking antibiotics compared to their housemates taking placebo, and positive results indicate greater diversity in the housemates taking placebo compared to the individuals taking antibiotics. For the control subjects, the bars represent the mean change in diversity amongst all control subjects. p-values were determined using the Mann Whitney U test, and the '*' represents p-values ≤0.05.
